# Bioinformatics procedure for investigating senolytic (anti‐aging) agents: A digital signal processing technique

**DOI:** 10.1002/agm2.12274

**Published:** 2024-01-08

**Authors:** Norbert Nwankwo, Ignatius Okafor

**Affiliations:** ^1^ University of Port Harcourt Port Harcourt Rivers State Nigeria; ^2^ University of Jos jos Plateau State Nigeria

**Keywords:** C‐terminal region 3, digital signal processing, DNA binding, D‐retro‐inverso, enhanced senescence 2, eterone 1, forkhead box protein O4, forkhead domain, senolytics, senomorphics

## Abstract

**Objective:**

Cell growth involves cell division. This stops after reaching a certain limit. Some cells become inactive and unable to undergo apoptosis (programmed cell death). These cells accumulate at sites of tissue damage or disease, thus accelerating aging. They are called senescent cells. Therapeutic interventions that can either eliminate senescent cells (senolytics) or suppress their harmful effects (senomorphics) have been developed. Senescence (aging) is caused by the inter‐ and intramolecular interactions between the domains of forkhead (FHD) and transactivation (TAD), as well as C‐terminal region 3 (CR3) and DNA binding (DBD). On the other hand, anti‐senescent/senolytic (anti‐aging) activities are achieved by disrupting these interactions with CR3‐ and forkhead box protein O4 (FOXO4)‐based peptides, such as ES2 and DRI, respectively. In this study, we use a computerized procedure based on digital signal processing to systematically analyze the inter‐molecular interactions between senolytics and their targets.

**Methods:**

Informational spectrum method (ISM) is engaged.

**Results:**

We obtained the sequences of the peptides from the interacting proteins of CR3 and FOXO4 and evaluated their ability to disrupt the inter‐molecular interactions between FOXO4 and DRI and CR3 and BDB, which are responsible for senescence (aging). Our results show that the peptides have different degrees of senolytic (anti‐aging) activity, depending on their affinity for CR3 and BDB, or FOXO4 and DRI. We found that enhanced senescence 2 (ES2) has a higher affinity for CR3 and BDB than FOXO4 and DRI, and that the interaction between CR3 and BDB is crucial for aging. Therefore, ES2 and other CR3‐based peptides are more potent senolytics than FOXO4‐based peptides. Our findings are consistent with previous studies and reveal new insights into the mechanisms of senescence and senolytics. ES2 is considered the best senolytic candidate, as it is 3–7 times more effective than DRI. We verified that ES2 has a weaker interaction with FOXO4 than CR3. However, the performance of DRI has been noted to depend on its intramolecular interactions and stability. Hence, intramolecular analyses using the digital signal processing‐based technique has become very vital and will follow.

**Conclusion:**

CR3‐based peptides are promising candidates for senolytic therapy. Senolytics are linear chains of amino acids that can target and eliminate senescent cells, which are cells that have stopped dividing and contribute to aging and age‐related diseases. By using this proposed, novel computerized technique that is based on digital signal processing, senolytics can be easily analyzed and optimized for their effectiveness and safety. This provides a more rational approach to enhancing our longevity and well‐being by offering interventions that can delay or reverse aging and insights that can advance the field of gerontology. This procedure also will compliment other approaches such as molecular stimulation, etc.

## Graphical abstract

An illustration of a novel simple approach to improving longevity using anti‐Aging (Senolytic) agents that are easily designed, developed and assessed using Fourier transformed‐based Digital Signal Processing technique.

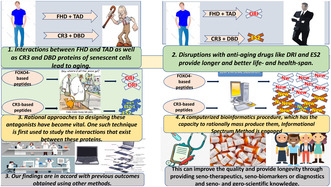

## 1 INTRODUCTION

Cell growth can involve cell division or differentiation. However, cells can stop growing due to natural limits or external stress, such as cancer or therapy. Some cells die and are removed (apoptotic), while others remain inactive and accumulate in the body (senescent). Senescent cells can cause inflammation and aging by secreting harmful factors, known as senescence‐associated secretory phenotype (SASP).^1^, ^2^, ^3^, ^4^, ^5^ SASP is associated with various diseases, such as diabetes,^6^ osteoarthritis,^7^ atherosclerosis,^8^ neurodegeneration, and cancer.^9^, ^10^ Senescent cells avoid death and aging by interacting between two proteins, forkhead box protein O4 (FOXO4) and p53,^2^, ^11^ which regulate cell fate. Specifically, the interaction between the C‐terminal region 3 (CR3) of FOXO4 and the DNA binding (DBD) of p53 as well as D‐retro‐inverso (DRI) and FOXO4 is crucial for senescence.^3^ Studies have shown that removing senescent cells from the body can improve health and lifespan, while adding senescent cells can have the opposite effect.^1^, ^2^, ^3^, ^4^ Aging is known to be driven essentially by cellular senescence and other factors,^1^, ^2^, ^11^ processes that impair cell function and growth. To explore how we can target and eliminate these harmful cells with senolytic agents, we used a bioinformatics approach to investigate the inter‐molecular interactions between senolytics and their protein partners. Intra‐molecular studies are also envisaged in future.

Some anti‐aging agents, such as Quercetin, Dasatinib, Fisetin, Navitoclax, and DRI, can clear the effects of senescent cells from our body either by removing the cells or inhibiting their activities. Those agents that remove the senescent cells are called senolytics.^1^, ^2^ They include DRI and enhanced senescence 2 (ES2). DRI is a reversed sequence of a part of FOXO4 protein, fused with a cell‐penetrating peptide (PPRRRQRRKKRG) from HIV‐TAT.^2^, ^11^, ^12^ Other agents that only antagonize senescent cells but do not remove them are called senomorphics.^1^ They include Rapamycin and its analogs, Rapalogs,^13^ Metformin, and Resveratrol.^1^

While DRI is a modification product, E1 (Eterone 1) as shown in Table 3, ES1 (enhanced senescence 1), ES2, ES2r1 (enhanced senescence 2 reverse 1), and ES2r2 (enhanced senescence 2 reverse 2) are seno‐therapeutic senescent peptides derived from the interacting domain of the BDB‐CR3^11^, ^12^, ^13^ This is recorded in Table 2. A clinical experiment using nuclear magnetic resonance (NMR) has shown that DRI not only restores frailty, fitness, lifespan, and healthspan, it also reinstates fur density and kidney dysfunction in aged mice as well as in mice with clinically induced aging pathologies.^2^, ^3^ ES2 has been identified to be 3–7 times more effective than the DRI.^12^

The two sets of peptides, namely FOXO4‐ and CR3‐based, are analyzed to re‐evaluate process, validate, and reaffirm the initial findings acquired through alternative methods. Our investigation focuses on two peptidic senolytics along with eight interacting peptides, as outlined in Tables 2 and 3. Additionally, this study incorporates an examination of molecular descriptors. To substantiate the selection of these molecular descriptors within the research context, we prioritize an initial assessment of the biophysical properties governing both inter‐ and intra‐molecular interactions. The interactions between forkhead box protein O4‐transactivation (FOXO4‐TAD) as well as C‐terminal region 3‐DNA binding (CR3‐DBD) that contribute to senescence and, consequently, aging, encompass both inter‐ and intra‐molecular interactions.^3^ Since this study specifically focuses on inter‐molecular interactions, only the molecular descriptor, the electron ion interaction potential (EIIP), that governs inter‐molecular interactions is employed. Complementary information from the intra‐molecular characteristics will later commence. Notably, there are more than 600 molecular descriptors, based on amino acid scales,^14^ that are readily available.^15^ Other alternative approaches for deriving suitable descriptors that regulate these interactions exist.

In this study, we reevaluated senolytics and their interacting domains using a digital signal processing approach, despite both being protein/peptide‐based molecules. This choice is attributed to the fact that proteins and peptides are well‐recognized for their linear arrangement of amino acids, which can be viewed as discrete components.^16^ This discrete nature makes them highly recommendable for analysis through digital signal processing (DSP) techniques, such as the discrete Fourier transform (DFT).^17^ Two DSP‐bioinformatics‐based physio‐mathematical techniques have seen widespread use in exploring bio‐functionalities. They are the informational spectrum method (ISM)^18^ and the resonant recognition method (RMM).^19^ In this study, we engaged ISM. Both RMM and ISM have played pivotal roles in uncovering the biological functions of numerous proteins, exceeding 1000 in total.^19^ These proteins include those associated with influenza,^20^ HIV,^21^, ^22^, ^23^ Anthrax,^24^ and Ebola.^23^ ISM continues to serve as the cornerstone for the development of biomedical tools and devices, such as the Computer‐Aided Drug Resistance Calculator^25^ and Phylogenetic tree.^26^ The engagement of ISM in this study serves a dual purpose—not only to reaffirm the interactions but also to deepen our comprehension of their significance in senescence. Moreover, it aims to pave the way for the creation of more effective seno‐ and gero‐therapeutic interventions.

Detailed information about both RMM and ISM can be found in previous works.^18^, ^19^, ^20^, ^21^, ^22^, ^23^, ^24^, ^25^, ^26^ A comprehensive description of the ISM procedure will be provided in the next section.

## 2 METHODS

The materials and the experimental procedure are described in this session.

### 2.1 Materials

The sequences of the senolytics and their target proteins are retrieved from Uniprot Database^27^ and other sources.^3^, ^11^ They are as shown in Tables 2 and 3. An example of a molecular descriptor (EIIP) is also shown in Table 1.

**TABLE 1:** values for the 20 essential amino acids on account of a molecular descriptor, electron ion potential (eiip).

AA	Value	AA	Value	AA	Value	AA	Value
A	0.0372	Q	0.0761	L	0.0000	S	0.0829
R	0.0959	E	0.0058	K	0.0371	T	0.0941
N	0.0036	G	0.0050	M	0.0823	W	0.0548
D	0.1263	H	0.0242	F	0.0946	Y	0.0516
C	0.0829	I	0.0000	P	0.0198	V	0.0057

### 2.2 Experimental procedure: Informational spectrum method (ISM)

ISM is applied to these agents using EIIP is as described below.

#### 2.2.1 Step 1: Translation of the protein sequences into numerical sequence (signals)

To begin, the alphabetical codes denoting the amino acids within the protein sequences under examination are replaced with numerical values corresponding to molecular descriptors. These molecular descriptors consist of 20 values, each reflecting the degree of involvement of one of the 20 essential amino acids in the interaction, spanning from physiochemical characteristics encompassing aspects like binding affinity, hydrophobicity, amphiphilicity, and various structural features such as helicity, Alpha (*α*), and Beta (*β*) properties.^22^ This transformation converts the protein sequences into numerical sequences or signals.

#### 2.2.2 Step 2: Zero‐padding of the numerical sequences (signals)

Following the DFT process principle, the numerical sequences, before undergoing decomposition with DFT, should maintain a consistent window length. Consequently, numerical sequences of proteins with shorter amino acid lengths undergo zero‐padding, meaning they are augmented with zeros until they reach the desired length. It is noteworthy that zero‐padding has been established to have no impact on the decomposition's results.^28^

#### 2.2.3 Step 3: Informational Spectrum: Decomposition of the Signal) using Discrete Fourier Transform

Fourier transform is fundamentally expressed as

(1) $$f\left(w\right)=\int_{\infty }^{\infty }f\left(t\right)e^{-\mathrm{jwt}}dt$$

e symbolizes exponential; *w* represents omega; *j* is called an imaginary complex number, ie, (−1)^1/2^ or √‐1; d*t* symbolizes changes in time.

Because these senolytic agents are peptides, amino acids in linear formation, they are converted into numerical sequences (signals) and decomposed with DFT^17^ to reveal the information embedded in them.

DFT is represented as:

(2) $$x\left(n\right)=\sum_{k=0}^{n=1}x\left(k\right)e^{-j2\mathrm{\pi kn}}$$

*n* is the discrete time index that runs from 0 to *n*−1, ie, 0, 1, 2,…, *n*−1; *k* is the discrete frequency index and runs 0 to *n*/2, ie, 0, 1, 2,…, *n*/2. This is because this process provides mirror image (symmetric) characteristics that remain the product of the Discrete Fourier Transform (DFT) processing. *x*(*k*) represents m member of the numerical series where *N* is the length of the numerical series. *X*(*n*) stands for the coefficient of the DFT.

The products of DFT application are a complex number consisting of real and imaginary, and absolute values.

Absolute values are usually utilized in determining the outcome of the DFT decomposition.

Absolute value is represented as:

(3) $$X\left(n\right)=\left[R\left(n\right)+I\left(n\right)j\right]$$

*n* = 1, 2…, *n*/2 while *R* and *I* represent the real and imaginary parts respectively; *j* is called an imaginary complex number, ie, (−1)^1/2^ or √−1 as explained above.

The degree of interaction, represented as the absolute spectrum^17^ is expressed as:

(4) $$S_{a}\left(n\right)=X\left(n\right)X^{*}\left(n\right)=\left|X{\left(n\right)}^{2}\right|$$

*S*_*a*_ signifies absolute spectrum of a given protein; *X(n)* represents the DFT coefficient of the signal; X*(n) is the conjugate, and *n* stands for numbers 1−*n*/2.

#### 2.2.4 Step 4: Common informational spectrum

This refers to the pointwise multiplication^17^ of all the derived FFT values of the protein's sequences investigated. This operation helps obtain one position of common interaction called consensus frequency (CF).^17^

Common informational spectrum is expressed as:

(5) $$C_{a}=\prod S_{\left(a\right)}\left(m\right)$$

*C*_*a*_ represents the absolute of the CS analysis; *m* = 1, 2,… M; M is the number of protein/peptide sequences engaged; ∏ is the pointwise multiplication symbol.

This position shows the highest amplitude after pointwise multiplication. The consensus frequency is represented as:

(6) $$\mathrm{CF}=\frac{\text{Position of Common Interaction}}{\text{Amino Acid length}}$$

Similarly, any frequency (*f*) of interest can be determined as:

(7) $$f=\frac{\text{Position of Interest}}{\text{Amino Acid length}}$$

By using both the CF and frequency of interest (*f*), degrees of interactions by any protein at any position can be determined. The results of this experiment are recorded in the subsequent session.

## 3 RESULTS

**FIGURE 1:**
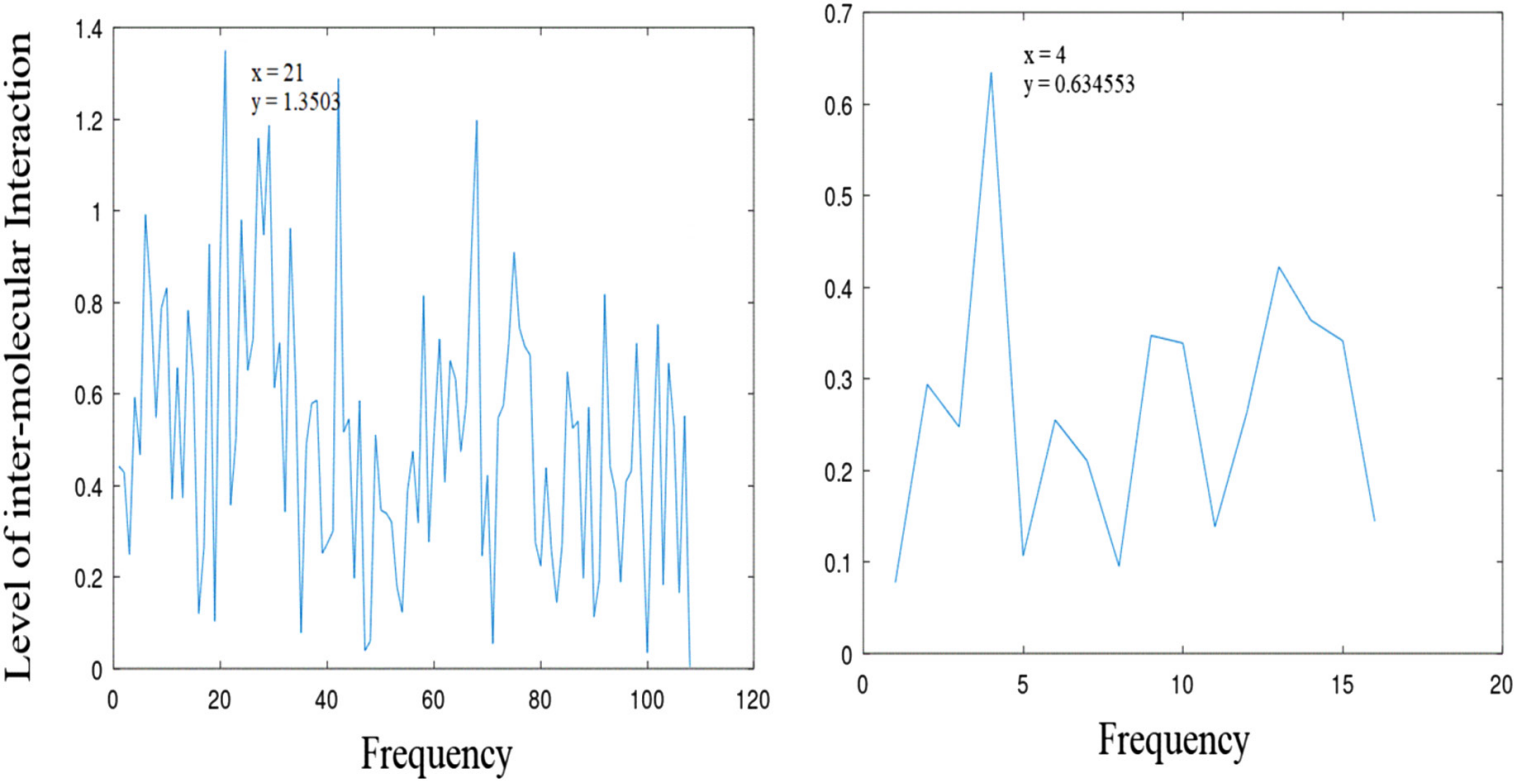
Informational characteristics of DNA binding domain (DBD) and CR3 showing maximum interaction (100%) at positions 21 (*f* = 0.960) and 4 (*f* = 0.125) with amplitudes (1.350 and 0.635), respectively.

**FIGURE 2:**
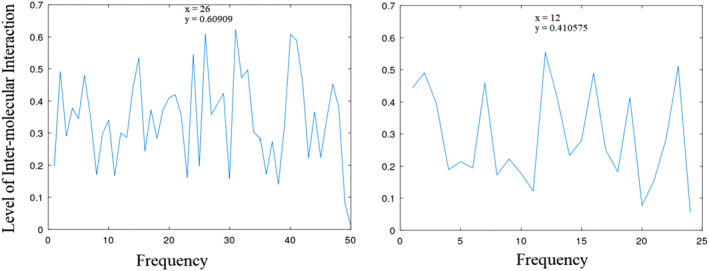
Informational characteristics of Forkhead Domain (FHD) and Transactivation Domain (TAD) showing amplitudes of 0.609 and 0.411 at positions 26 (*f* = 0.257) and 12 (*f* = 0.245), respectively.

**FIGURE 3:**
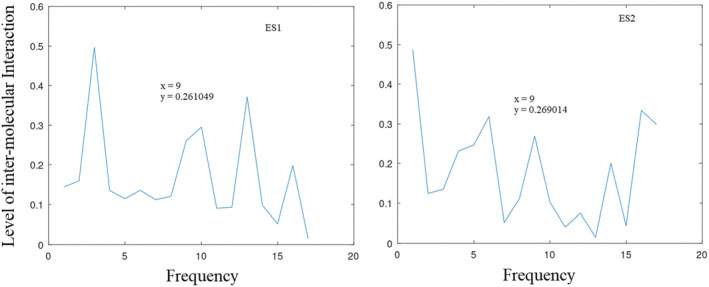
Informational characteristics of ES1 and ES2 showing amplitudes of 0.261 and 0.269 at position 9 (*f* = 0.257); see Table 4.

**FIGURE 4:**
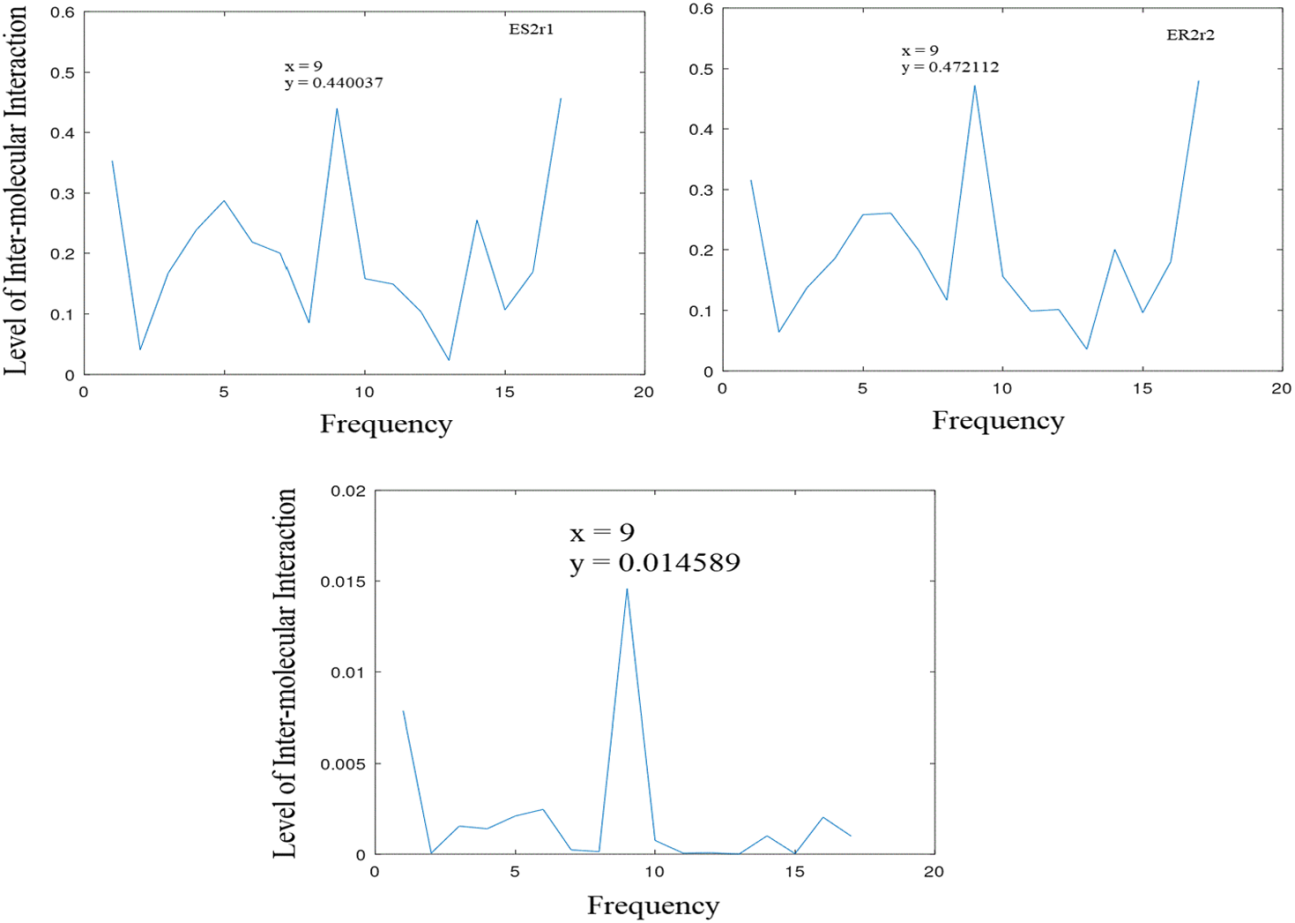
Informational characteristics of ES2r1, ES2r2, and their common informational characteristics, which includes ES1 and ES2 showing maximum interaction at positions 9 (*f* = 0.257), respectively.

**FIGURE 5:**
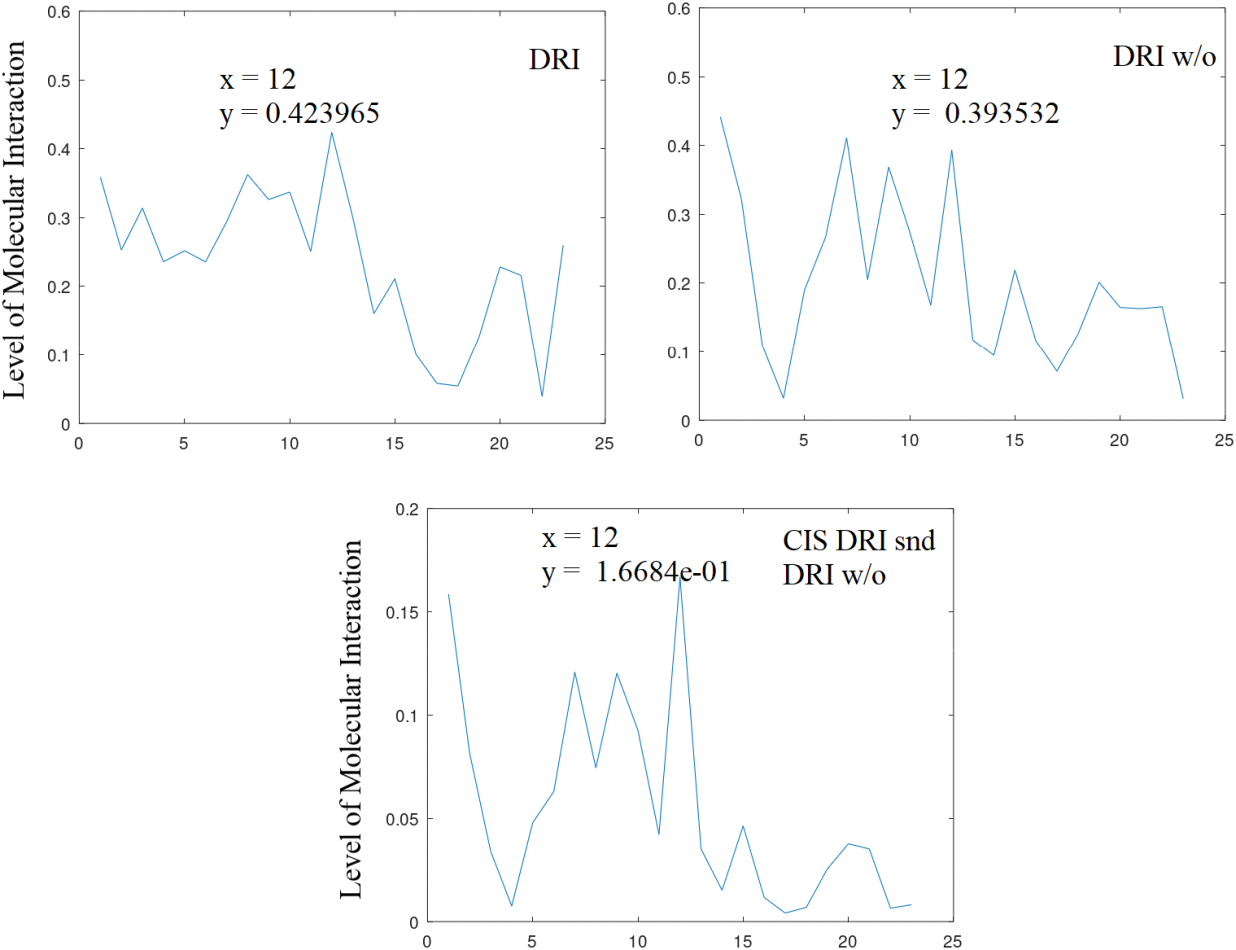
Informational characteristics of DRI‐with and DRI‐without the cell penetrating peptides (zero padded and processed) showing amplitudes of (0.424 and 0. 394) at position 12 (*f* = 0.261), respectively.

**FIGURE 6:**
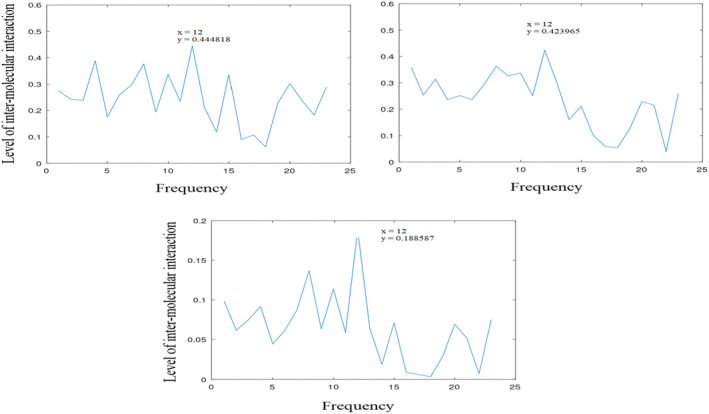
Informational characteristics of DRI and E1 (DRI with 4 mutations) and their common informational characteristics showing amplitudes of (0.423965, 0.444818, and 0.188587) at position 12 (*f* = 0.261), respectively.

Our findings are presented across Tables 2, 3, 4, 5 and Figures 1, 2, 3, 4, 5, 6. Table 1 provides insights into senescence and senolytic activities, contingent on the involved peptide, expressed as inter‐molecular (binding) interactions attributed to the 20 essential amino acids (EIIP). Tables 2 and 3 detail the peptides under scrutiny. Table 2 outlines CR3 and CR3‐based peptides along with their sequences (ES2r1, ES2r2, ES1, and ES2), while Table 3 documents FOXO4‐related peptides and their interacting proteins (TAD, FHD, DBD, DRI, and E1). Table 4 presents the informational spectral values for CR3‐related peptides, displaying their amplitudes (indicating degrees of interactions). Table 5 records the informational spectral data for FOXO4‐based peptides. Table 6 offers a summary of the findings, including amino acid interaction positions/frequencies, interaction degrees (percentages) in individual plots, and their comparative contributions.

**TABLE 2:** Sequences of CR3 and CR3‐based senolytics.

Peptide	Sequences
CR3	DLDLDMYMENLECDMDNIISDLMDEGEGLDFN
ES2	PRKGGRRRRAWGRRRRRRRRRRRRRRAPRKRLTLA
ES2r1	PRKGGRRRRAWGRRRRRRRLRRRLRRAPRKRLTLA
*ES2r2*	PRKGGSRRRAWGRRRYRRRLRRRLRRAPRKRLTLA
ES*1*	YGRKKRRQRRRYGRKKRRQRRRYGRKKRRQRRR

**TABLE 3:** Sequences of FOXO4‐DRI, variant (e1), and their interacting proteins.

Peptide	Sequences
TAD	PLSQETFSDLWKLLPENNVLSPLPSQAMDDLMLSPDDIEQWFTEDPGPD
FHD	GSRRNAWGNQSYAELISQAIESAPEKRLTLAQIYEWMVRTVPYFKDKGDSNSSAGWKNSIRHNLSLHSKFIKVHNEATGKSSWWMLNPEGGKSGKAPRRRA
BDB	SSSVPSQKTYQGSYGFRLGFLHSGTAKSVTCTYSPALNKMFCQLAKTCPVQLWVDSTPPPGTRVRAMAIYKQSQHMTEVVRRCPHHERCSDSGLAPPQHLIRVEGNLRVEYLDDRNTFRHSVVVPYEPPEVGSDCTTIHYNYMCNSSCMGGMNRRPILIITLEDSSGNLLGRNSFEVRCACPGRDRRTEEENLRKKGEPHHELPPGSTKRALPNNT
DRI	LTLKKEPASEIAQSILEAYSQNGWANRRSGGKRPPPRRRQRRKKRG
DRI without CPP	LTLKKEPASEIAQSILEAYSQNGWANRRSGGKRP
E1	GRKKRRQRRRPPPRKGGSRRRAWGNQRYARLIRQAIESAPEKRLTL

**TABLE 4:** Informational spectral values of CR3‐based peptides.

Peptide	Sequences
CR3	0.077 0.294 0.247 **0.635** 0.107 0.255 0.210 0.095 0.347 0.339 0.138 0.263 0.422 0.364 0.341 0.144
ES1	0.145 0.150 ***0.497*** 0.136 0.115 0.136 0.112 0.121 **0.261** 0.295 0.091 0.093 0.372 0.098 0.052 0.199 0.015
*ES2r1*	0.353 0.041 **0.168** 0.239 0.288 0.219 0.201 0.085 **0.440** 0.158 0.149 0.104 0.023 0.255 0.107 0.170 0.457
*ES2r2*	0.316 0.064 0.137 0.186 0.258 0.261 0.199 0.117 **0.472** 0.156 0.099 0.101 0.035729 0.201 0.096 0.181 0.480
*ES2*	0.487 0.125 0.135 0.232 0.247 0.319 0.052 0.112 **0.269** 0.103 0.041 0.075 0.015 0.201 0.044 0.334 0.299

*Note*: Bold and italics presentations in the tables, etc are used to point out the positions of interactions and highlight their relevance.

**TABLE 5:** Informational spectral values of FOXO4‐based peptides.

Peptide	Sequences
DRI	0.359 0.253 0.314 0.236 0.252 **0.** 0.294 0.363 0.326 0.337 0.251 **0.424** 0.300 0.160 0.211 0.100716 0.058 0.054 0.126 0.229 0.216 0.039 0.260
*E1*	0.274 0.242 0.238 0.389 0.175 0.260 0.297 0.377 0.194 0.337 0.234 **0.445** 0.211 0.119 0.335 0.090 0.107 0.062 0.228 0.303 0.237 0.183 0.288
TAD	0.443 0.491 0.394 0.189 0.214 0.194 **0.459** 0.172 0.222 0.178 0.122 **0.554** 0.411 0.233 0.280 0.490 0.250 0.182 0.414 0.077 0.155 0.285 0.510 0.055
*DRI without CPP*	0.442 0.322 0.109 0.0319 0.190 0.267 0.411 0.205 0.369 0.275 0.168 **0.394** 0.117 0.095 0.219 0.116 0.071 0.126 0.202 0.165 0.163 0.166 0.031

*Note*: Data for Forkhead Domain (FHD) and DNA Binding Domain (DBD) are too large and are not displayed here. Plots are provided. The boldness signifies position of interaction.

**TABLE 6:** Summary of the entire findings 2: Comparative contributions.

Peptide	Position of interaction (frequency)	Percentage interaction	Comparative contributions
DBD	21 (0.960)	100	
CR3	4 (0.125)	100	
ESI	3 (0.999)	100	
ES2r1	**9 (0.257)**	96	**0.440 (93%)**
ES2r2	**9 (0.257)**	100	**0.472 (100%)**
ES2	**9 (0.257)**	52	**0.269** (57%)
ES1	**9 (0.257)**	55	**0**.**261 (55%)**
FHD	**26 (0.257)**	**98**	
TAD	**12 (0.245)**	100	
DRI	**12 (0.261)**	71	**0.424** (95%)
E1	**12 (0.261)**	100	**0. 445 (100%)**
DRI without CPP	**9 (0.265)**	100	**0.394** (89%)

*Note*: The boldness signifies position of interaction.

Figure 1 displays the informational characteristics of the DBD and CR3 (DBD data omitted due to its extensive nature). Notably, DBD exhibits its highest interaction at position 21 (*f* = 0.960) with an amplitude of 1.3503, while CR3 demonstrates maximum interaction at position 4 (*f* = 0.125) with an amplitude of 0.635. Moving to Figure 2, it highlights the informational features of FHD and TAD, showing amplitudes of 0.609 and 0.411 at positions 26 (*f* = 0.960) and 12 (*f* = 0.125), respectively. Lastly, Figure 3 presents the informational attributes of ES1 and ES2, revealing amplitudes of 0.261 and 0.269 at positions 9 (*f* = 0.257), respectively.

Figure 4 illustrates the informational spectral characteristics of ES2r1, ES2r2, and their CIS. This CIS analysis includes those of ES1 and ES2, as shown in Figure 2. The CIS demonstrates a maximum amplitude of 0.146 at position 9 (*f* = 0.257).

It has been observed that the FOXO4‐p53 interaction leads to senescence (aging) through the interaction between FHD and TAD, and these interactions can be interrupted by DRI and ES2, providing senolytic (anti‐aging) activity. To enhance the efficacy of DRI, it is fused with the cell penetrating peptide (CPP). To assess the impact of CPP on senolytic activities, both the naive DRI peptide and DRI, which embodies the CPP, are evaluated. These results are presented in Figure 5. To facilitate a comparative assessment of the two peptides with unequal lengths, the shorter sequence is first zero‐padded, processed, and the CF determined. As shown in Figure 5 and Table 6, DRI demonstrates 95% senolytic (anti‐aging) activity through inter‐molecular interaction at position 12 (*f* = 0.261), while DRI without CPP shows 89% activity at a similar frequency, ie, position 12 (*f* = 0.261). The fusion with CPP results in higher senolytic activity, justifying the inclusion of CPP.

In the design of DRI, four amino acid changes were made on E1. To investigate the impact of these changes on inter‐molecular interactions, both DRI and its variant E1 are reassessed and recorded in Figure 6. DRI and E1 exhibit amplitudes of 0.424 and 0.449 at position 12 (*f* = 0.261), respectively. The CIS of both DRI and E1 records 0.189. These results are displayed in Figure 6. A comparative analysis of this outcome (Table 6) reveals that E1 demonstrates greater inter‐molecular activity (100%) than DRI (95%). This suggests that intra‐molecular interactions may play a more significant role in senolytic properties, as previously suggested by H. Le.

In summary, as indicated in Table 6, all the senolytics, both CR3‐based and DRI‐related peptides, share a common position of interaction (common frequency, CF) not only among themselves but also with their interacting proteins. While the CR3‐based peptides, namely ES2r1, ES2r2, ES1, and ES2, share the position (*f* = 0.257) among themselves, they also interact with FHD and TAD at positions (*f* = 0.257) and (f = 0.245), as well as with DRI (*f* = 0.261), E1 (*f* = 0.261), and DRI without CPP (*f* = 0.261). However, at this position, ES2r2 and ESrr12 exhibit a higher interacting potential (100% and 93%, respectively) than ES1 and ES2 (55% and 57%, respectively).

In line with the ISM principle, sharing the same frequency indeed indicates interaction, as previously demonstrated. An analogous scenario exists in the context of HIV infection progressing to AIDS, which results from the interaction between HIV viruses and the host CD4. These proteins have been observed to share frequencies of 0.035 and 0.037, respectively.^22^

Another noteworthy finding is that ES1, a three repeat of the cell‐penetrating peptide, displays a notable propensity to interact with CR3. ES1 exhibits the highest amplitude (100%) at position 3 (*f* = 0.999), an interacting position closer to those of DBD (*f* = 0.960) and CR3 (0.125) than FHD (*f* = 0.257) and TAD (*f* = 0.245). This suggests that ES1 could be a promising candidate for disrupting the CR3‐DBD interaction, pending complementary assessments with intra‐molecular interactions. The interaction between CR3 and DBD is pivotal in senescence, and its disruption holds the potential for anti‐aging effects.

## 4 DISCUSSION

The results of this computational assessment seem to align with a clinically verified discovery that there exists an interaction between the forkhead domain and the transactivation domain (TAD) of p53. This is evident as they both share a common interaction frequency point. TAD demonstrates maximum interaction (100%) at *f* = 0.245, while FHD exhibits a strong interaction of 98% at *f* = 0.257 (Table 6). However, DRI, which is responsible for anti‐senescence (senolytic or anti‐aging) activity through the disruption of the FOXO4‐p53 interaction, demonstrates a 100% interaction at approximately the same frequency (0.261) with both FHD and TAD. DRI, which is a d‐amino acid retro‐reverse sequence derived from the binding domain of the FOXO4‐p53 interaction and designed by Baar et al.^2^, ^3^, ^11^, aimed for enhanced efficacy. The retro‐reversal of the sequence, coupled with fusion with the CPP, has shown in this study to significantly contribute to its anti‐aging effectiveness. Furthermore, this study highlights the beneficial impact of the CPP fusion, aligning with findings obtained through alternative methods.

Remarkably, ES2, although selected as the candidate, demonstrates 3–7 times greater activity than DRI. It is essential to highlight that ES2, while highly active, shows a lower affinity for FOXO4‐p53. This phenomenon may be attributed to what H. Le et al. suggested: that the drugability or suitability of ES2 as a drug candidate is potentially influenced by intra‐molecular interactions and stability, warranting further exploration.

That our findings indicate that ES1, a repeat CPP peptide, shares the highest inter‐molecular interaction position with CR3 and DBD demonstrates that it holds promise as a CR3‐based senolytic candidate, assuming the intra‐molecular features can provide similar outcomes. It is worth noting that the interaction between CR3 and DBD, which is known to facilitate the FOXO4‐p53 interaction,^3^ is already acknowledged as a pivotal factor in the senescence process.

## 5 CONCLUSION

Interaction between FHD and TAD, as well as CR3 and DBD, are known to contribute to senescence (aging). However, interruptions by CR3‐ and FOXO4‐based peptides such as ES2 and DRI, respectively, have been identified as providing anti‐senescence (anti‐aging) activities. The clearance of senescent cells using senolytics or the inhibition of their activities through senomorphics is a recognized therapy that enhances both lifespan and healthspan (longevity). The involvement of these peptides in this health management was made possible by the works of H. Le et al. and Baar et al.^1^, ^2^, ^3^, ^11^ Baar et al. introduced the d‐amino retro‐reverse sequence of the FOXO4‐p53 interacting domain known as DRI, while H. Le et al. modeled CR3‐based peptides like ES1, ES2, ES2r1, and ES2r2, identifying ES2 as a candidate. Understanding the interactions governing these senolytics and their interacting proteins has become crucial in designing and developing more effective seno‐therapeutic interventions and contributing to gero‐scientific studies. This necessitates the use of simple, computerized and bioinformatics‐based approaches as provided here.

As discrete structured biomolecules, peptides, which are amino acids in linear formation, are easily analyzed using digital signal processing techniques. Utilizing this bioinformatic and computerized procedure has helped improved healthspan and longevity to humanity through the development of seno‐therapeutics, seno‐biomarkers, and gero‐scientific studies. Here, we therefore present a simple, novel technique that will expand assessments and development of senolytics. This approach will compliment other procedures like molecular simulation, etc. The validity and authenticity of the procedure rest on the technologies the technique, Fourier transform (FT), has earlier provided. Radar is a technology that detects, locates, tracks, recognizes objects like airplanes in the air. It provide flight safety. Speech detector is another device that uses voice recognition to grant authorized accesses to restricted area. These technologies are both founded on same technique employed here, Fourier Transform.^17^

## AUTHOR CONTRIBUTIONS

Concept, design, data mining, data processing, analysis, and presentation by Norbert Nwankwo. Analysis, design, and presentation are also carried out by Ignatius Okafor.

## FUNDING INFORMATION

Not Applicable.

## CONFLICT OF INTEREST STATEMENT

Not Applicable.
